# Symposium: Reproductive technology and the conceptualization of the biological clock

**DOI:** 10.1016/j.rbms.2022.02.001

**Published:** 2022-03-02

**Authors:** Anindita Majumdar

**Affiliations:** Indian Institute of Technology Hyderabad, India

## Introduction

The biological clock as an idea and idiom is closely associated with the emergence and impact of new reproductive technologies. Fertility treatments have contributed to the popular and medical discourse of the biological clock by making it a signature image of declining and resurgent fertility (Bledsoe, 2002, Friese et al., 2006, Lock, 2007). The increasingly prominent role of new reproductive technologies in assisting and extending all aspects of the reproductive cycle have both heightened the importance, and intensified the contradictions, of the ‘biological clock’ analogy.

By definition, the idea of the biological clock promotes a futuristic outlook: seeking to arrest reproductive decline, or anticipate its outcomes (Majumdar, 2018), is the primary function of this increasingly common reference point to a presumed biological limit-point. Reproductive technologies become part of the rhetoric and practice of this anticipatory rhetoric of loss, challenging us to subvert it ‘naturally’ (Lock, 2007, Sanabria, 2016). The biological clock at once embodies and promotes the imperative for technology to counter depleting (feminine) nature, and its rhythmic cycles (Bear, 2014), while at the same time referencing the fallacy of being able to control nature, despite the promise of ‘assisting’ reproduction (Franklin, 2002).

This Symposium issue aims to highlight the tensions in these widespread imaginings around the biological clock as it is made, remade and unmade in the context of reproductive biomedicine. In this attempt, we are interested in looking at how technology facilitates particular notions of controlling the reproductive body, while simultaneously highlighting the risks of both technological assistance and embodied temporality. Taking into account several important strands of emerging research, we offer a critical engagement with the notion of the biological clock as it becomes enmeshed in the conceptualization of the body and time within assisted reproduction.

## Time is money

The ticking clock is more than a metaphor in both **Lucy Van de Wiel** and **Heather Jacobson’s** contributions. Van de Wiel’s research on the ‘financialization of infertility’ explores how venture capitalists have begun to invest in proactive fertility insurance through ‘company-sponsored fertility benefits’. This new financialization of fertility risk promotes new engagements among women working in global corporations, which encourage ‘proactive’ management of one’s fertility while thus enforcing the need for fertility insurance to safeguard the fulfilment of reproductive hopes, plans and desires. At the same time, for the American surrogates in Heather Jacobson’s article, the amount of time spent in each IVF-surrogacy arrangement means a negotiation of other ‘life clocks’. The money spent on managing what Jacobson calls the ‘ART clock’ weighs heavily not only intended parents – who may have to pursue multiple IVF cycles until their surrogate becomes pregnant—but on surrogates as well. Here, time *is* money as surrogates must learn to navigate childcare and emotional investment in the intended parents' lives with the uncertainty of IVF cycles, as seen in repeated embryo transfers and hormone cycles, miscarriages and other exigencies.

## Ageing = decline?

Eggs and oocytes become markers of ageing and decline in **Nolwenn Bühler**’**s** analysis of fertility preservation and reproductive technologies. Bühler embarks on an archaeology of cellular manipulation within reproductive science in the desire for longevity. In order to subvert reproductive decline, Bühler suggests that age and ageing become more complicated as well. At the same time, in **Anindita Majumdar’s** study, assisted reproduction becomes an important node of conceptualizing post-menopausal reproduction, especially in facilitating reproduction amongst those past their reproductive prime. In rural North India, assisted reproductive technology is part of the attempt to overturn the narrative of declining reproduction, by facilitating pregnancies amongst ageing women, while at the same time reinforcing socially debilitating practices of son preference in the name of intergenerational longevity.

Together, our biological clock articles reveal the importance of continuing to engage critically with this ubiquitous, complicated and highly paradoxical idiom, which, having become an emblem of our time, is also a measure of its mixed messages about the future of fertility care, preservation and hope.

## Declaration

The author reports no financial or commercial conflicts of interest.
